# Coordinated surface activities in *Variovorax paradoxus *EPS

**DOI:** 10.1186/1471-2180-9-124

**Published:** 2009-06-12

**Authors:** W David Jamieson, Michael J Pehl, Glenn A Gregory, Paul M Orwin

**Affiliations:** 1 Department of Biology and Biochemistry, University of Bath, Bath, BA2 7AY, UK; 2 Department of Biology, California State University, San Bernardino, 5500 University Pkwy, San Bernardino, CA, 92407, USA; 3 Current address: Loma Linda University, School of Dentistry, Loma Linda, CA, USA

## Abstract

**Background:**

*Variovorax paradoxus *is an aerobic soil bacterium frequently associated with important biodegradative processes in nature. Our group has cultivated a mucoid strain of *Variovorax paradoxus *for study as a model of bacterial development and response to environmental conditions. Colonies of this organism vary widely in appearance depending on agar plate type.

**Results:**

Surface motility was observed on minimal defined agar plates with 0.5% agarose, similar in nature to swarming motility identified in *Pseudomonas aeruginosa *PAO1. We examined this motility under several culture conditions, including inhibition of flagellar motility using Congo Red. We demonstrated that the presence of a wetting agent, mineral, and nutrient content of the media altered the swarming phenotype. We also demonstrated that the wetting agent reduces the surface tension of the agar. We were able to directly observe the presence of the wetting agent in the presence and absence of Congo Red, and found that incubation in a humidified chamber inhibited the production of wetting agent, and also slowed the progression of the swarming colony. We observed that swarming was related to both carbon and nitrogen sources, as well as mineral salts base. The phosphate concentration of the mineral base was critical for growth and swarming on glucose, but not succinate. Swarming on other carbon sources was generally only observed using M9 salts mineral base. Rapid swarming was observed on malic acid, d-sorbitol, casamino acids, and succinate. Swarming at a lower but still detectable rate was observed on glucose and sucrose, with weak swarming on maltose. Nitrogen source tests using succinate as carbon source demonstrated two distinct forms of swarming, with very different macroscopic swarm characteristics. Rapid swarming was observed when ammonium ion was provided as nitrogen source, as well as when histidine, tryptophan, or glycine was provided. Slower swarming was observed with methionine, arginine, or tyrosine. Large effects of mineral content on swarming were seen with tyrosine and methionine as nitrogen sources. Biofilms form readily under various culture circumstances, and show wide variance in structure under different conditions. The amount of biofilm as measured by crystal violet retention was dependent on carbon source, but not nitrogen source. Filamentous growth in the biofilm depends on shear stress, and is enhanced by continuous input of nutrients in chemostat culture.

**Conclusion:**

Our studies have established that the beta-proteobacterium *Variovorax paradoxus *displays a number of distinct physiologies when grown on surfaces, indicative of a complex response to several growth parameters. We have identified a number of factors that drive sessile and motile surface phenotypes. This work forms a basis for future studies using this genetically tractable soil bacterium to study the regulation of microbial development on surfaces.

## Background

*Variovorax paradoxus *is a ubiquitous, aerobic, gram negative bacterium present in diverse environments [1,2]. This organism, originally classified in either the genus *Alcaligenes *or *Hydrogenomonas*, has been associated with a number of interesting biotransformations, including atrazine degradation [3], nitrotyrosine assimilation [4], and mineralization of acyl-homoserine lactone signals [5]. Recently, the hydrogen gas oxidation growth strategy of *V. paradoxus *has been implicated in plant growth promotion [6], as part of the rhizosphere consortium with nodulating diazotrophs. This microorganism was also recently identified as a member of methylotrophic community in the human oral cavity [7]. In spite of its ubiquity, and a wealth of interesting metabolic capacities, relatively little has been published on the physiology of *V. paradoxus*.

The morphology of bacterial colonies is an often described feature used in identification of isolates from diverse sources. It is frequently observed that colony morphology is a crucial indicator of strain variation [8], which has been used productively at least since Griffith's experiments with pneumococci. Organisms such as *Myxococcus xanthus *have been studied extensively and productively to understand differentiation processes on a surface[9]. Gliding, swarming, swimming, and twitching motility have been categorized and catalogued in many species [10]. More recently, it has become clear that the complex communities of bacteria forming a colony on an agar plate can be used as a model system for studying growth physiology. The alterations in colony morphology that are observed in mutants of *Vibrio cholerae *correlate with differences in biofilm structure[11], and *Pseudomonas aeruginosa *retrieved from continuous biofilm cultures were shown to be genetically diverse, contrasting with the monoculture input to the system [12]. Additionally, recent work in model systems of surface growth has shown that motility on agar plate surfaces, swarming, twitching, gliding, and swimming, is strongly affected by the plate environment [13]. Swarming as an agar plate physiology has been studied for many years in *Proteus *species, which grow and swarm very rapidly in culture [14]. It has also been studied in several other model systems, such as *Bacillus subtilis *[15], *Serratia liquifaciens *[16], *Pseudomonas aeruginosa *[17], and several other pathogenic and environmental isolates (for review see [18]). Swarming motility is intimately involved in the virulence of many important pathogens [19], but is also a standard physiological response to environmental conditions [20]. Interestingly, although swarming motility in particular is observed in a wide array of microorganisms, the effect of nutrient sources on swarming has only been studied in a few systems. Carbon sources associated with swarming were identified in *Salmonella *species, showing that different strains responded to different nutrient classes [21]. In *P. aeruginosa*, certain amino acids can stimulate swarming motility [22]. Carbon source dependence in *P. aeruginosa *has only been examined under a few circumstances, showing that *P. aeruginosa *does not swarm on succinate in FAB medium [22,23]. This work also addressed the role of nutrients in the physiology of flow cell biofilms, suggesting that surface roughness is related to nutrient sources. Based on other work, it has been suggested that salt concentration also plays a role [24], probably by altering the water availability at the agar surface. Surface wetting has been observed to impact swarming in *Salmonella*, with flagella playing roles in wetness detection and motility through the activity of FlgM [25].

Over the past decade, the study of microbial biofilms has grown exponentially (for review see [26,27]). The biofilm lifestyle is now universally acknowledged as the dominant form of microbial growth in the environment, ranging from desert crusts to biofilms on hospital catheters [27]. Several model systems have been utilized to examine the genetics of biofilm formation, and the gram-negative *Pseudomonas aeruginosa *has become a particularly well studied system. The study of this and other model biofilms has made clear some of the salient features of this lifestyle, such as attachment, growth, maturation, and detachment [28-30]. Other microorganisms have also been extensively studied, but most of the effort has understandably focused on biofilms of medical interest, such as urinary tract pathogens [31], dental or periodontal disease associated bacteria [32], enteric pathogens [33], and gram positive cocci associated with catheter and nosocomial wound infections [34,35]. For those working on environmental biofilms, much of the focus has been on biofouling, with *Shewanella*, which is capable of oxidizing iron containing surfaces in a marine environment, as a model system [36]. Finally, plant-root and epiphytic biofilms have become an interest for microbiologists interested in crop protection or crop enhancement, as microbial community structures have demonstrated repeatedly their influence on plant health and agricultural yield [37].

In this report we examine swarming motility and biofilms formed by the aerobic soil bacterium *Variovorax paradoxus*. We demonstrate that swarming is a fundamental behavior of this microorganism, and examine the effect of Congo Red, an acidic dye that disrupts flagellar function, on swarming. In this context we observe the production of a wetting agent, possibly a surfactant. We examine carbon sources, nitrogen sources and water content in the agar as key factors in swarming motility. We also examine the biofilms formed under similar nutrient conditions in a 96-well polystyrene microtiter plate assay, as well as the role of fluid shear on biofilm formation by *V. paradoxus *attached to a glass surface. Finally, we observe that dense, structurally complex biofilms are formed readily by this microorganism in continuous culture. We suggest that *V. paradoxus *EPS is a valuable additional model of complex coordinated surface behavior in proteobacteria, and can be used to understand the role of this microbial population in soil and rhizosphere environments. These surface behaviors and the signals that drive them are likely related to the nutrient cycles driven by plant root exudates in the rhizosphere.

## Methods

### Bacteria used

*V. paradoxus *strain EPS was cultivated from the soil in the Land Lab at CSU San Bernardino. *Pseudomonas aeruginosa *PAO1 was obtained from the *Pseudomonas *Genetic Stock Center (East Carolina University), S17-1 was obtained from ATCC (ATCC# 47055).

### Swarming motility

Swarming motility was routinely assayed using freshwater (FW) base medium (Table 1) [5] solidified with 0.5% agarose (Low EEO, Fisher Scientific), supplemented with 1:1000 dilutions of a trace metal mixture (ATCC TM-S) and a vitamin mixture (ATCC TV-S). This medium was buffered to pH 7 with 5 mM MOPS. Additional swarming assays were performed using M8/M9 minimal media [22](Table 1, Difco) supplemented with the same constituents. In all swarming motility assays, triplicate samples on an individual petri dish were measured, and diameters recorded to the nearest millimeter in each measurement.

**Table 1:** Composition of minimal media used (per liter)

M8/M9 salts	FW medium
0.2% w/v carbon source^a^	0.2% w/v carbon source^a^
0.1% w/v nitrogen source^a, b^	0.1% w/v nitrogen source^a^
3.0 g KH_2_PO_4_	0.2 g KH_2_PO_4_
8.18 g Na_2_HPO_4 _dihydrate	
0.5 g Mg_2_SO_4_heptahydrate	0.15 g Na_2_SO_4_
	0.4 g MgCl_2 _hexahydrate
0.15 g CaCl_2_dihydrate	0.1 g CaCl_2 _dihydrate
0.5 g NaCl	1.0 g NaCl
5 mM MOPS	5 mM MOPS
Trace Vitamins (ATCC)	Trace Vitamins (ATCC)
Trace Minerals (ATCC)	Trace Minerals (ATCC)

^a^In media with casamino acids as sole C and N source, 0.1% w/v was used

^b^M8 medium is defined as M9 using alternative N sources

### Congo Red Inhibition

FW based plates as described above were made containing 0.2% sodium succinate and 0.05% NH_4_Cl as carbon and nitrogen sources. The plates were supplemented to varying concentrations with Congo Red (0.1% stock solution, filter sterilized). The plates were allowed to dry for 4d before inoculation. The plates were inoculated from an overnight culture grown in FW-succinate-NH_4_Cl broth. The inoculum was pelleted by centrifugation and resuspended at an OD595 of 1.0 in sterile water. A 5 μl spot was inoculated on the plates and allowed to dry for at least 1 h before growth at 30°C. A set of plates was incubated in a glass dish containing a wet paper towel to maintain heightened humidity. Colony diameter measurements and images were collected over a 72 h period post inoculation from plates inoculated in triplicate. For imaging purposes, additional plates were inoculated with single drops centrally.

### Drop collapse assay

The wetting agent zone was visualized and marked. A 0.01% methylene blue solution was made in sterile water, and a 2 μl drop was applied to the agar surface and the wetting agent surface. The response was immediately photographed.

### Nutrient requirements for Swarming

Alternative carbon sources (maleic acid, malic acid, sucrose, benzoate, maltose, mannitol, d-sorbitol) were tested at 0.2% w/v, with other constituents as stated above, with ammonium chloride as sole nitrogen source. Casamino acids were tested as sole carbon and nitrogen source at 0.1% w/v final concentration. Water and agarose were autoclaved, cooled to approximately 50°C, and supplemented with other components prior to plate pouring. Succinate was used as the carbon source for determination of nitrogen source dependence. NH_4_Cl, (NH_4_)_2_SO_4_, glycine, methionine, histidine, tryptophan, tyrosine, cysteine, and arginine were all tested as potential stimuli for swarming, at 0.05% final concentration (w/v). All amino acids used were the L-forms (Fisher Scientific). Colony diameter measurements and images were collected over a 72 h period post inoculation.

### Microtiter biofilm cultures

Cultures were inoculated from overnight growth in M9 based broth containing succinate as sole carbon source, and NH_4_Cl as sole nitrogen source. For nitrogen or carbon source tests, the overnight culture was pelleted and resuspended in the nutrient medium of interest at a 1:100 dilution from the original culture, and dispensed in replicates (6 for each condition) in the wells of a microtiter dish. The edge wells were filled with sterile water, and the lid was coated with Triton X-100 diluted in 70% EtOH to prevent condensation [38]. Plates were prepared in duplicate, for assay at 24 h and 48 h. At 24 h, one plate was washed 3× with water, and stained for 15 m with 1% crystal violet (CV). This stained plate was washed thoroughly with water to remove all free CV, and dried overnight. The CV was resolubilized in 95% EtOH and the absorbance was measured at OD595 in a Thermomax microtiter spectrophotometer (Molecular Devices). The liquid media were aspirated from the second plate, and replaced with fresh media for growth over the second 24 h period. After 48 h it was stained with CV and read as described for the 24 h plate. In all experiments, a negative control well for each nutrient condition and time was also read. The nitrogen and carbon sources tested for effects on swarming motility were likewise examined for effects on biofilm formation.

### Biofilm reactor

Batch biofilm experiments were performed in Nalgene autoclavable plastic jars with holes drilled in the lid using a 1 1/4 inch bit. Clean glass slides were held in place using cut rubber stoppers, and the chamber was filled with growth media. The entire batch reactor was autoclaved prior to inoculation. For batch experiments with media replacement, the lid and slides were transferred to a fresh autoclaved media jar for further growth. A stir bar was placed in the chamber prior to autoclaving for stirred batch experiments. The CDC bioreactor (Biosurface Technologies, Bozeman, MT) was also used for stirred batch and continuous culture experiments. All culture experiments were performed using 0.5 g/L YE broth as the growth medium. The CDC bioreactor is capable of utilizing a total of 24 coupons for sampling, on eight individual polystyrene coupon holders. For these experiments, the initial reactor setup contained four coupon holders loaded with glass coupons. The entire reactor is autoclaved prior to use, with unattached hoses covered with foil. The full biofilm chamber with four coupon holders was filled with 0.5 g/L YE to just above the level of the top coupons (~350 ml) prior to autoclaving. Additional coupon holders with polycarbonate chips (Biosurface technologies) were autoclaved and used to replace the experimental samples to maintain the appropriate mechanical shear conditions.

### Stirred Batch Culture

An overnight culture of the test bacteria was grown at 30°C with shaking at 200 rpm overnight in 0.5 g/L YE. Overnight culture was added to the biofilm reactor at a 1:500 dilution (using an approximate culture volume of 350 ml), All cultures were stirred at 150 rpm using a magnetic stir plate (Cimarrec) at room temperature. Glass slides or glass coupons were removed from the chamber aseptically, and stained with crystal violet or with the BacLight (Invitrogen, L-7012) kit reagents to identify live and dead bacterial cells in situ.

### Stirred Continuous Culture

Cultures were inoculated as described for batch cultures. All initial cultures and starter cultures were grown in 0.5 g/l YE. After 18 h of batch culture incubation, one coupon holder was removed, and replaced with an autoclaved coupon holder containing polycarbonate chips. The removed coupons were examined for biofilm growth (batch culture). At this point, the peristaltic pump (Carter Manostat, Cole-Parmer) was started, providing continuous flow of 0.5 g/L YE broth at 1.9 ml/min (residence time 185 m). A diagram of the CDC reactor system as it was used for this study is available from the manufacturer at http://www.biosurfacetechnologies.com. After 24 h of culture under these conditions, one coupon holders was again replaced aseptically, and examined by epifluorescence microscopy. After 48 h of continuous culture, all remaining biofilm coupons were removed and examined by epifluorescence microscopy.

### Viability Staining

The biofilms on disks in batch culture were examined by epifluorescence microscopy using the BacLight viability staining kit (L-7012, Invitrogen). Staining was performed by covering the inward face of the glass coupon in the stain mix in a sterile 12 well plate, and washing with sterile water after the appropriate time. Five minutes with a concentrated stain mix (1.5 μl of each stain per ml) was found to be sufficient. Stained glass coupons were mounted on cleaned glass slides, and observed by epifluorescence microscopy using an Axioplan 2 microscope (Carl Zeiss, NY) equipped with appropriate filter sets (41002, 41017, Chroma Technologies), and an Xcite-120 illuminator (Exfo Life Sciences, Ontario, Canada). Images were captured using an SBIG 1402-XME (Santa Barbara Instruments, Santa Barbara, CA mounted on a 1× c-mount adapter, with a 0.2 second exposure. The monochrome images were captured using the CCDops software supplied with the camera. Captured images were merged using ImageJ http://rsb.info.nih.gov/ij/. The camera ccd was cooled maximally for all fluorescence imaging (20°C below ambient). Whole image contrast and brightness enhancement was used to optimize for publication only.

### Visible light imaging

Still images from swarming plates and time lapse movies were captured with a CoolSnapFX (Roper Scientific) cooled ccd camera using ImagePro MC Express on a Zeiss Axioplan 2. Biofilms were examined using 1% Crystal Violet as a simple stain. Color images were captured using a Kodak DC290 digital camera, using the Kodak image capture software provided. Macroscopic colony images and wetting agent images were collected using a Fuji FinePix 5700 digital camera. Colonies were photographed using a black velvet cloth to damp reflection. To capture images of the wetting agent, the plate was illuminated using diffuse reflected light, and angled to capture the refractive quality of the layer. For all microscopy, calibration images were captured with all microscope lenses of a stage micrometer, and Image J was used for measurement and scaling.

## Results

### Swarming motility

**Figure 1:**
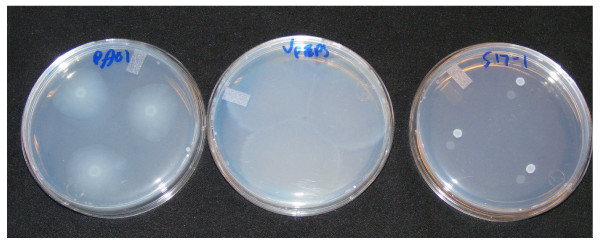
***Variovorax paradoxus *displays swarming motility**. Swarming plates with glucose and casamino acids inoculated with drops of *P. aeruginosa *PAO-1 (**A**), *V. paradoxus *EPS (**B**), or *E. coli *S17-1 (**C**).

Our laboratory developed a swarming agar plate based on previous growth and swarming experiments in *V. paradoxus *and *P aeruginosa*. Our swarming agar used for initial studies used 0.5% agarose to solidify the plate, the freshwater media (FW) base previously used by Leadbetter and Greenberg [5], with 0.2% glucose as a carbon source. Previous work in *P. aeruginosa *PAO1 suggested that casamino acid (CAA) supplementation is necessary [39], so 0.1% CAA was added to this media, along with NH_4_Cl, as nitrogen source. Spot inoculation of *V. paradoxus *EPS, *P. aeruginosa *PAO1, and *Escherichia coli *S17-1 on this swarming agar was performed (Fig 1). *V. paradoxus *EPS and *P. aeruginosa *PAO1 show strong swarming activity on this media, although the patterns are strikingly different. *E. coli *S17-1 shows no swarming, but robust growth, on this medium. Using gradient plates, we determined that glucose was not a suitable substrate for swarming on FW based media using NH_4_Cl as nitrogen source (not shown).

### Inhibition of Swarming with Congo Red

**Figure 2:**
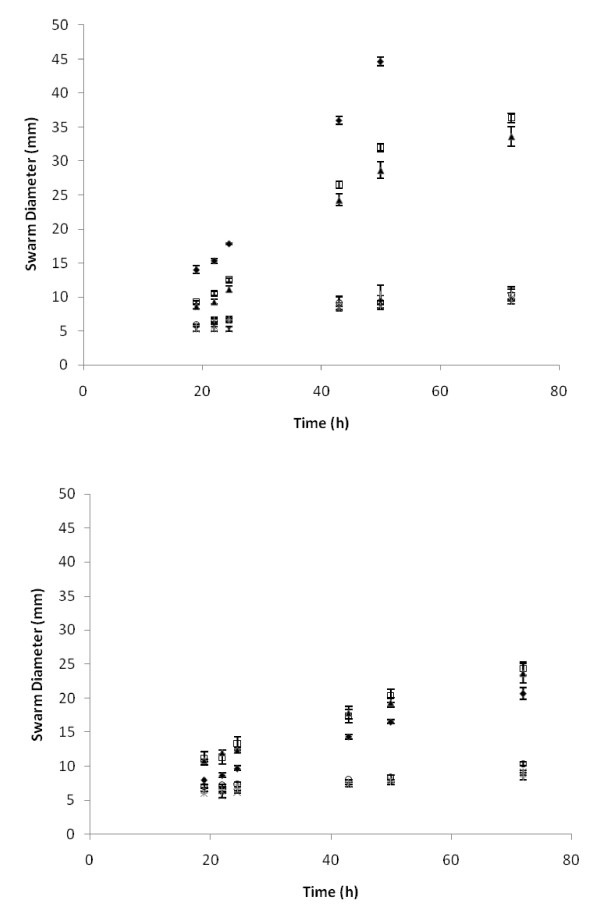
**Swarming of *V. paradoxus *EPS is inhibited in a dose dependent manner by the presence of Congo Red in the agar**. Plates containing doses of Congo Red ranging from 1–1000 μg/L were incubated at 30°C either A) under ambient atmospheric humidity or B) in a humidified glass dish. Symbols in both panels: No CR (black diamond), 1 μg/L CR (open square), 10 μg/L CR (filled triangle), 50 μg/L CR (×), 100 μg/L(*), 500 μg/L CR (open circle), 1000 μg/L (+). Swarm diameter measured in triplicate, reported as mean ± SEM.

**Figure 3:**
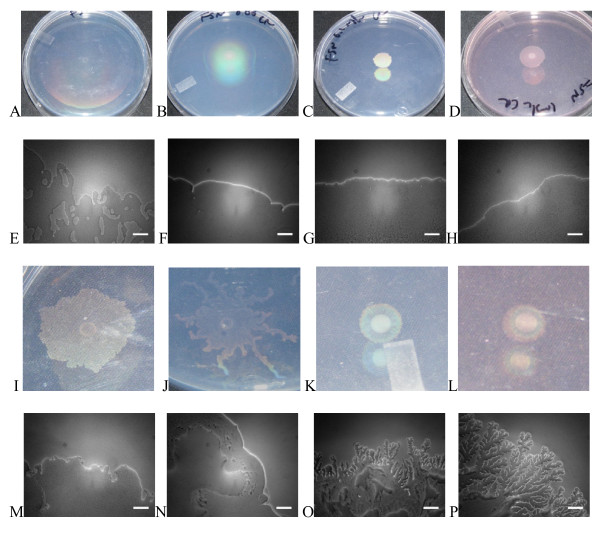
**Humidity affects response to Congo Red swarming inhibition**. A-D) gross morphology of *V. paradoxus *EPS on plates incubated at 30°C on media containing 0, 10,100, and 500 μg/L CR after 48 h. E-H) Edge images from the same culture conditions at 24 h. I-L) gross morphology of 48 h cultures on identical media incubated at 30°C in a humidified chamber. M-P) edge images from the humidified chamber incubated cultures at 24 h. Scale bar = 25 microns.

Swarming requires the presence of flagellar activity, which is inhibited by Congo Red (CR) [40]. Supplementing plates with ≥ 50 μg/L CR had a strong inhibitory effect on the swarming phenotype (Fig 2). The colony did expand in diameter over a 48 h period under CR conditions, but at a much lower rate, consistent with simple growth based expansion. The microscopic analysis of the colony edges (Fig 3E–H) shows that the morphology of the edge differs markedly on plates containing CR. Robust growth of *V. paradoxus *EPS was observed under all CR treatment conditions (Fig 3A–D).

### Role of a wetting agent in swarming

**Figure 4:**
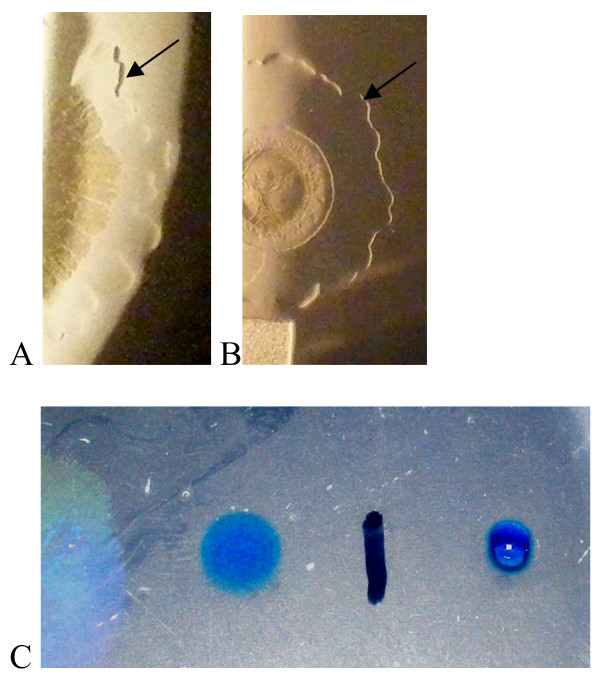
**A wetting agent is present beyond the edge of the swarm**. Colony photography using reflected light (A, B) illustrating the presence of a wetting agent (arrows) preceding the spreading colony on (A) FW medium with succinate and NH_4_Cl as C, N source. B) Colony spread is limited by 500 μg/L CR, but wetting agent spreads as above. C) Drop collapse assay using dilute methylene blue solution showing the reduced surface tension in the wetting agent zone (left of the black line).

Swarming is dependent on the presence of a wetting agent, which can be seen spreading on the plate (Fig 4A, B). Wetting agent is observed spreading well in advance of the colony on media containing inhibitory levels of CR (Fig 4B). The wetting agent is evident on plates without CR during the first 2d of growth (Fig 4A), and the wetting agent reduces the surface tension of the agar plate, as shown using a qualitative water drop collapse assay (Fig 4C).

### Impact of humidity on swarming

When the incubation of the plates was performed in a humidified chamber, the swarming rate under all permissive conditions was reduced (Fig 2B). The physiology of the swarm was significantly altered by humid incubation (Fig 3). For morphological analysis of humidified colonies, magnified images were used, which are not directly comparable in size to the non-humidified samples. In the absence of CR, the gross morphology of the swarms (Fig 3A, I) differed markedly. Swarming on CR in the humidified incubator was characterized by macroscopic tendrils at low concentrations (Fig 3J), which were not seen during swarming under non-humidified conditions (Fig 3B). At higher CR concentrations, the gross morphology did not differ due to humidification (Fig 3C, D, K, L), but the edges viewed microscopically were sharply altered, with a pronounced branching pattern evident that increased with CR dose (Fig 3M–P). No branching of this sort was observed at any concentration of CR under non-humidified conditions (Fig 3E–H). No wetting agent was observed preceding the swarms on humidified plates, regardless of CR treatment (not shown).

### Swarming motility on different carbon sources

**Figure 5:**
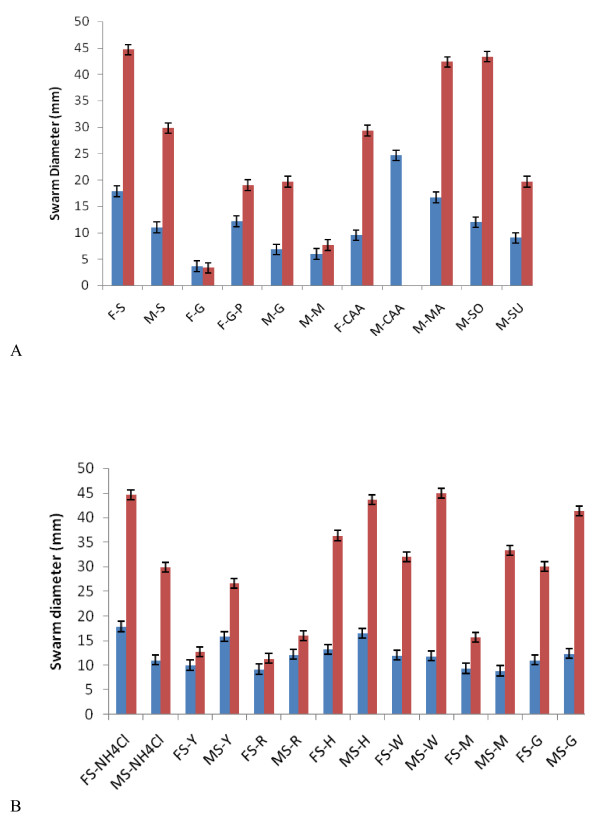
**Nutrient dependence of swarming motility**. A) Swarm diameter at 24 h (blue bars) or 48 h (red bars) using several carbon sources on FW (F) or M9 (M) base. F/M-S = succinate, F/M-G = glucose, F-G-P = glucose + 2 mM phosphate buffer (pH7), M-M = maltose, F/M-CAA = casamino acids (C+N), M-Ma = malic acid, M-So = sorbitol, M-Su = sucrose. * indicates that swarms merged by 48 h. B) Swarm diameter at 24 h (blue bars) or 48 h (red bars) using several nitrogen sources on FW (F) or M9 (M) base. All swarms measured in triplicate, with error in all cases ± SEM.

**Figure 6:**
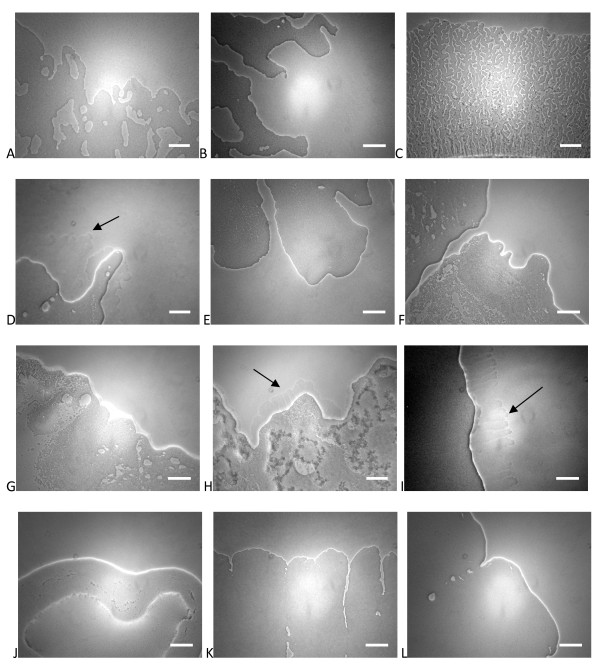
**Edges of swarms are affected by nutrients, basal medium**. Swarming edge images after 24 h on a variety of media. FW base medium was used for (A, B, D, J, K, L) with M8/M9 base medium used for the other panels. Succinate is the C source in all panels except B (glucose) and C (maltose). For growth on FW-glucose, 2 mM sodium phosphate buffer (pH 7) was added. NH_4_Cl was the N source in (A-C), with alternative N sources methionine (D, E), arginine (F), tyrosine (G, J), tryptophan (H, K), and histidine (I, L). Arrows point to extruded material from swarm edges under certain conditions. Scale bar = 25 microns.

**Figure 7:**
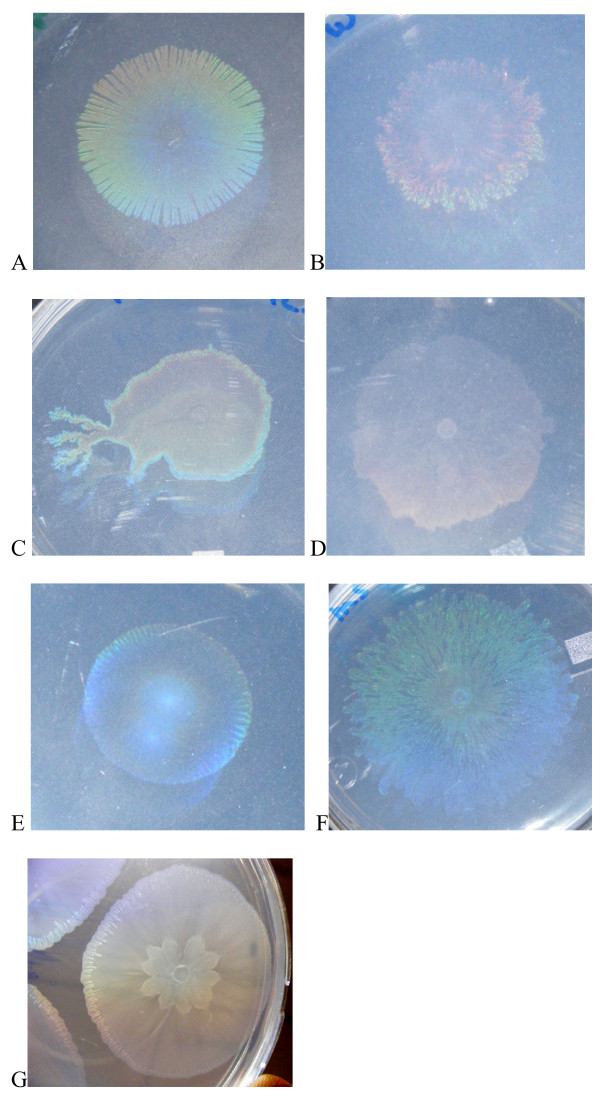
**Gross swarm morphology is affected by nutrients, basal medium**. Colony morphologies after 1d on A) FW-succinate-NH_4_Cl and B) FW-casamino acids. C) After 3d on FW-succinate-methionine, a "rare branch" phenotype was observed. D) Slower swarming on M9-succinate-tyrosine was characterized by a less well defined swarm with altered structure. Stark differences in extent and form of swarming were observed on E) FW-succinate-tryptophan and F) M9-succinate-tryptophan. G) After an extended incubation, swarms on FW-succinate-NH_4_Cl display a mutually repellent morphology with distinct internal and external edges.

Experiments were undertaken to determine what carbon sources could induce swarming on two different basal media (Table 1) containing NH_4_Cl as sole nitrogen source. On the FW base medium, only casamino acids (as sole C and N) and succinate supported robust swarming, with a minimal level of swarming observed on d-sorbitol and very delayed minimal swarming on malic acid (Table 2). When 2 mM sodium phosphate buffer (pH 7) was added to FW glucose media, growth in liquid media was restored (not shown), and swarming was similar to M9 glucose (Fig 5A). On M9 based media, however, all carbon sources except maleic acid and sodium benzoate supported swarming motility (Table 2). Over a 48 h period, rapid swarming on d-sorbitol, malic acid, and succinate was observed (Fig 5A). Swarming was slower on glucose and sucrose, and slowest on maltose (Fig 5A). Swarming on maltose was characterized by long branches that failed to merge over long distances (Fig 6C). Swarming on other carbon sources on M9 resulted in similar edge phenotypes to the succinate edges. When multiple swarms were developing on a single plate, a repulsion effect was observed, such that the two growing swarms did not merge (Fig 7G). Cultures grown on either basal medium with CAA as sole C-source were strikingly disorganized (Fig 7B), and merged together on the plate (not shown). These later experiments demonstrated that our initial swarming result was due entirely to the inclusion of CAA in the medium (Fig 7B).

**Table 2:** Swarming and Planktonic Growth of *V. paradoxus *EPS

	Broth Growth (24 h)		Swarming^a^		Biofilm
**Carbon Sources**	M9	FW	M9	FW	M9

Casamino acids	++	++	++	++	+++

Glucose	++	+/-	+	+/-	++

Succinate	++	++	++	++	+++

Benzoate	++	++	-	-	+/-

Maltose	++	-	+*	-	+/-

Sucrose	++	-	+	-	+

d-Sorbitol	++	-	++	+/-	++

Maleic acid	+	-	-	-	+/-

Mannitol	++	-	++	-	+

Malic acid	++	-	++	+/-	++

**Nitrogen Sources (with Succinate)**					

NH_4_Cl	++	++	++	++	+

NH_4_SO4	++	++	++	++	+

Tryptophan	++	+	++	++	+

Histidine	++	+	++	++	+

Methionine	++	-	+	+	+

Cysteine	-	nd	Nd	Nd	nd

Tyrosine	++	-	+	+	+

Arginine	++	nd	+	+	+

Glycine	++	-	+/-	+	+

* swarming was slower with distinct edge (Fig 3, 4)

### Swarming motility on different nitrogen sources

When succinate was used as carbon source, all single amino acids tested were permissive for swarming on FW minimal base as well as M8 base (Table 2). When the swarm diameters were measured at 24 h and 48 h, a pattern similar to the carbon source experiments was observed (Fig 5B). Rapid swarming was observed on NH_4_Cl, tryptophan, histidine, and glycine (Fig 5B). Swarming at a slower pace was evident on tyrosine (Fig 7D), arginine, and methionine. Cultures on methionine had a "rare branch" phenotype (Fig 7C) that was different from other nitrogen sources The swarm progressed more rapidly on M9 than on FW base in all of these cases, in contrast with NH_4_Cl, and the tryptophan swarms were strikingly different in appearance (Fig 7E, F). An extruded tendril was clearly evident on plates containing methionine, histidine, and tryptophan as sole N-source, under certain basal media conditions (Fig 6D, H, I arrows).

### Nutrient dependence in biofilms

**Figure 8:**
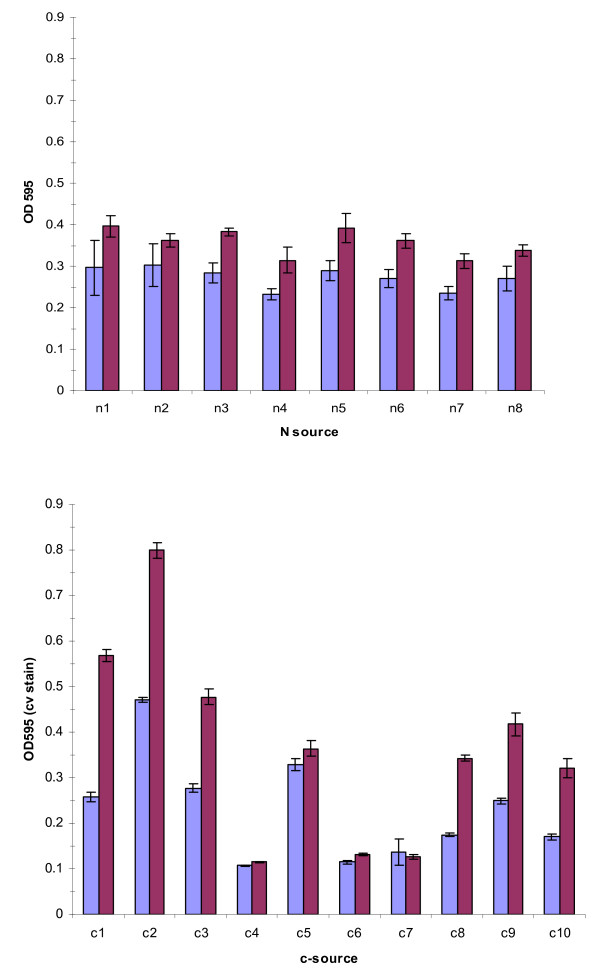
**Nutrient dependence of batch biofilm formation**. A) Biofilm formation with succinate as carbon source is not dependent on nitrogen source. N1 = methionine, N2 = tyrosine, N3 = tryptophan, N4 = NH_4_SO_4_, N5 = glycine, N6 = arginine, N7 = histidine, N8 = NH_4_Cl. B) Biofilm formation on variable carbon sources with NH_4_Cl as nitrogen source. C1 = glucose, C2 = casamino acids, C3 = succinate, C4 = maleic acid, C5 = d-sorbitol, C6 = maltose, C7 = benzoate, C8 = mannitol, C9 = malic acid, C10 = sucrose. In both instances measurements were taken after 24 h (blue bars) and 48 h (red bars). Error is computed as ± SEM.

Biofilms were grown in microtiter dishes at 30°C with shaking. Identically inoculated plates were grown for 24 or 48 h, with media replacement at 24 h. The biofilm was examined by staining with crystal violet. With succinate as sole carbon source, dense biofilms were formed after 48 h on all the nitrogen sources tested (Fig 8A). However, carbon source tests demonstrated significant alterations in biofilm formation, with NH_4_Cl used as the nitrogen source in all cases (Fig 8B). The thickest biofilms were formed in media containing casamino acids as sole carbon source. Student's unpaired t-tests were used to determine the significance of raw biofilm formation differences between cultures as compared to succinate or glucose. In all cases, all c-sources were significantly different in biofilm level compared to either succinate or glucose after 48 h, indicating a strong dependence of biofilm formation on carbon source. No significant differences in biofilm formation were observed when cultured on succinate with varying n-sources.

### Batch biofilms

**Figure 9:**
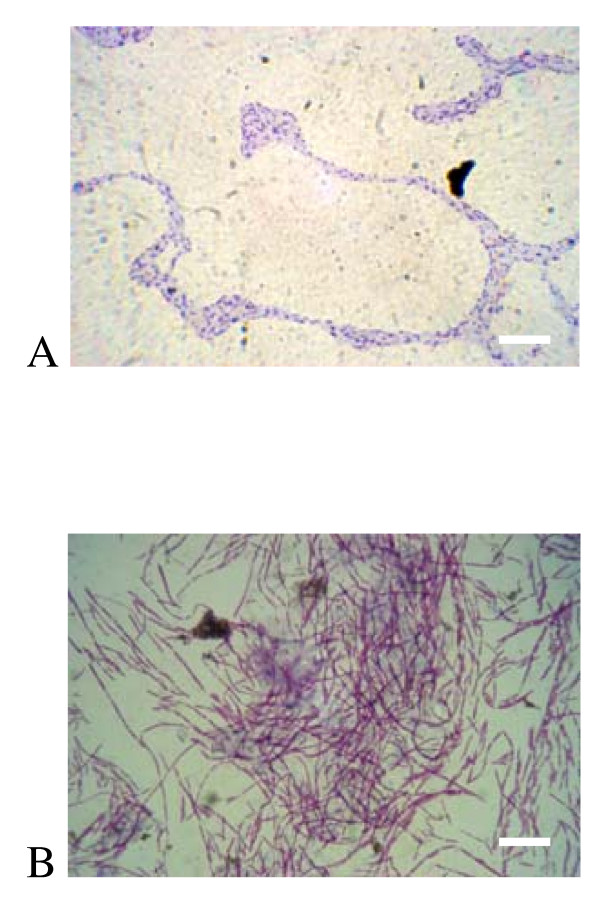
**Static and Stirred batch biofilms**. A) A static biofilm grown for 48 h in a Nunc one-well plate shows characteristic biofilm forms near the air-broth interface when stained with 1% crystal violet. B) *V. paradoxus *EPS from a stirred batch bioreactor on a glass slide show a strong propensity toward filamentous morphology. Both images at 1000× magnification. Scale bar = 10 microns.

Static batch biofilms display the traditional morphological markers associated with this growth morphology, including dense formations near the air-water interface, the characteristic honeycomb structure (Fig 9A). Biofilms were also grown under shear stress on glass slides in a stirred reactor, under batch conditions. Stirred batch biofilms in 0.5 g/L YE demonstrated filamentous growth, but the overall growth on the surface was sparse, with little accumulation of characteristic biofilm towers (Fig 9B).

### Chemostat biofilm culture

**Figure 10:**
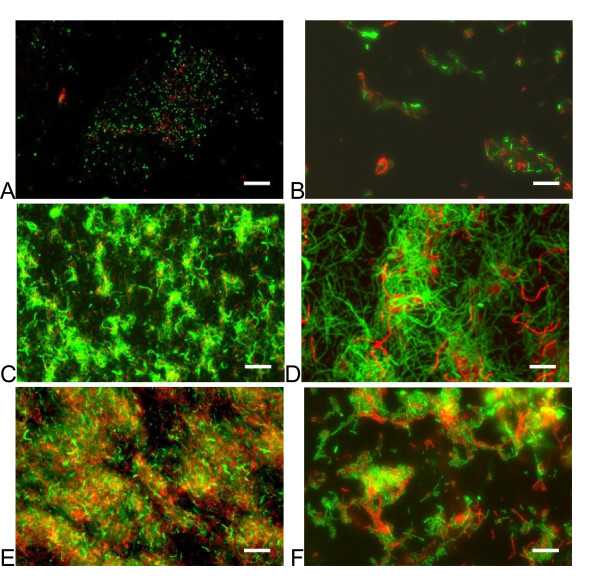
**Biofilms cultivated in a CDC stirred biofilm reactor**. *V. paradoxus *EPS was cultured from a broth inoculum for 18 h under stirred batch conditions (A, B), followed by 24 h (C, D) or 48 h (E, F) under continuous flow conditions (2 ml/min). BacLight staining with PI (red, dead cells) and Syto9 (green, live cells). 100×, scale bar = 100 microns (A, C, E). 400×, scale bar = 25 microns (B, D, F).

*V. paradoxus *EPS was inoculated into a Biosurface Technologies CDC biofilm reactor and grown as a batch culture for 20 h (Fig 10A, B). Continuous culture for 2d after this initial batch phase resulted in the formation of a dense, filamentous biofilm (Fig 10C–H). Staining with the BacLight system (Invitrogen) showed a mixed population of live and dead cells at all stages of development. At higher magnification, the filamentous structures of the developing biofilm are readily apparent, and filaments that stain with propidium iodide, indicating dead cells, are particularly strongly evident.

## Discussion

The environmental bacterium *Variovorax paradoxus *is involved in a number of important processes, such as promoting plant growth and remediation of xenobiotics. Our work with the *V. paradoxus *strain EPS demonstrates that this strain is capable of coordinated surface behaviors in laboratory culture. The behaviors we've examined in this report are the development of a swarm on defined high water activity (low agarose content) media and the formation of biofilms on several abiotic surfaces.

We have examined the capacity of this organism to move across a solid surface, and identified the motility demonstrated as swarming. We utilized agarose as the solidifying agent in our media, at 0.5% w/v, based on previous swarming analyses [39] and auxotrophy studies in our lab showing that *V. paradoxus *EPS utilizes organic components of bacteriological agar as nutrients (not shown). The motility was shown to require flagellar activity (Fig 2, 3), and to involve the production of a chemically uncharacterized wetting agent (Fig 4). The presence of 1–3 flagella per cell on swimming *V. paradoxus *has been noted in previous work, and is cited as a defining characteristic of this taxon [41]. We identified these flagella in broth cultures of our strain (not shown). In the recently released draft sequence of *V. paradoxus *S110, genes encoding flagellar components have been identified (Han et al, http://genome.ornl.gov/microbial/vpar_s110). Based on these data along with our experimental results, we feel justified in labeling the surface motility observed as swarming motility.

Our experiments allow some insights into the mechanism of *V. paradoxus *EPS swarming. Swarming is inhibited by Congo Red with a threshold value of 50 μg/L, consistent with the inhibition of the function of a single flagellum. However, we do not know if the inhibition by CR is complete, and will need to evaluate flagellar mutants to confirm these results. In staining experiments, we found no evidence for a hyperflagellated swarmer cell. This is similar to reports using *P. aeruginosa *in swarming studies, where the cell morphology was elongated, but polar localization of the flagella was maintained [22]. The production of the wetting agent is inhibited when the bacteria are incubated in a humidified chamber (Fig 3), and the swarming rate is reduced under those conditions (Fig 2). This indicates that the wetting agent is critical for a full swarming response. Some motility is observed in the cultures with inhibitory levels of CR present, which may be consistent with an alternative motility such as sliding motility [18]. The observed branching pattern on plates incubated in a humidified chamber with inhibitory concentrations of CR is consistent with an alternative mode of surface movement, driven by increase production of hydrophilic exopolysaccharide, or alternatively by the matrix absorbing water from the air, and thereby increasing the spread of the colony. The observed edge is consistent with increased colony water content, and the absence of a wetting agent to decrease the surface tension of the agar. Further investigation of this possibility is necessary. Although surfactants such as rhamnolipid [39], serrawettin [42], and surfactin [15] have been identified as critical components of swarming, in at least one case there is evidence that the wetting agent is not a surfactant [43]. We are currently in the process of isolating and identifying the *V. paradoxus *EPS wetting agent using biochemical and genetic means.

The swarms display the polarity observed in many species, with repellent signals inhibiting the merging of adjacent swarms (Fig 7G). Under certain nutrient conditions, such as use of CAA as sole C and N source, swarms merge readily (not shown). A similar response was seen when tryptophan was used as sole N source, suggesting that this amino acid is involved in the phenotype. An explanation for this response may be related to the production of exopolysaccharides (eps), which may be responsible for the fluid flow in the expanding swarm. The force that drives swarm expansion may be generated by flagellar activity as well as the accumulation of a hydrophilic eps that flows out from the dense center of the swarm. Increased formation of eps may result in "overflow" of the swarm, where the edge cannot stop fast enough to prevent the mixing of adjacent swarms. Alternatively, the wetting agent composition may be altered under certain conditions, leading to the observed changes in motility and swarm structure. Recent work has supported the idea that swarms respond to repellent signals based on the detection of specific signals encoded in the *ids *gene cluster in *Proteus mirabilis *[44]. It remains to be seen if a similar mechanism is common in other bacteria, and whether the mechanism is conserved.

The interrelationship of nutrient sources and basal medium had a strong impact on swarming motility. Rapid swarming was observed using several carbon sources on M8 medium, but only succinate and CAA supported swarming on FW based medium. The transport of glucose (and some other sugars) is limited by low levels of phosphate in FW medium. When FW medium is amended with phosphate, swarming is restored, along with higher growth yields in vitro (not shown). Even in the presence of phosphate, however, swarming is more robust on succinate than glucose. This result contrasts with results from *P. aeruginosa *[23]. However, the minimal media used in these experiments are different, and this comparison merits further study. It remains to be determined what other factors might be involved in reduced swarming rates on glucose when phosphate is not limiting. The most striking carbon source based difference was in response to maltose, where the rate of swarming and the structure of the swarm differed sharply with observations on other carbon sources. Comparison of the swarm edge on maltose (Fig 7C) with the swarm edge on succinate inhibited by CR and humidified (Fig 3O, P), is suggestive of the possibility that the lack of wetting agent may be partially responsible for this phenotype.

The results with CAA, along with previous work on swarming in *P. aeruginosa *led us to wonder about amino acids as sole nitrogen sources in the context of swarming. Several of the amino acids tested were able to support robust growth and swarming with succinate as a carbon source, while others were conducive to less robust swarming. We did not identify any amino acids that supported growth but not swarming. Obviously, however, our testing was not exhaustive, and future work will examine the remaining amino acid substrates. Our results show substantially different response patterns to those seen previously in *P. aeruginosa *PAO1 [22]. With the exceptions of histidine and glycine, which were conducive to swarming in both organisms, all of the amino acids which we tested did not support *P. aeruginosa *PAO1 swarming. It should be noted here that in this instance the same basal medium (M8) was used, although we tested an additional basal formulation. This may relate to the differences in the ecological niches for these organisms, and the predominance of amino acids in plant root exudates. The specific composition of the organic material in the source soil for *V. paradoxus *EPS has not been determined. The presence of very thin tendrils beyond the edge of the swarm is discernable by phase contrast microscopy on several amino acid nitrogen sources (Fig 6, arrows). This extruded substance does not appear to correlate with swarming rate, and is distinct from the wetting agent that we see macroscopically. Based on time-lapse video microscopy using wild-type and mutant *V. paradoxus *EPS strains [see Additional file 1] the extruded material is likely to be an extracellular polysaccharide that allows for the rapid movement of the swarm outward from the inoculums (Pehl et al, manuscript in preparation). The experiments performed here allow for a clear set of alternative hypotheses concerning the development of *V. paradoxus *EPS swarms. The availability of growth limiting substrates may be the key factor, or some particular nutrients may have a more direct effect through specific signals. This can be directly tested in growth experiments using combinations of nutrients, as well as by analysis of mutant population swarming characteristics. Experiments of both of these types are either planned or ongoing.

Biofilm formation in M9 based medium was robust with succinate as carbon source, regardless of nitrogen source, over 24 and 48 hour batch culture. Dense biofilms were also present with several other carbon sources, notably d-sorbitol, glucose, malic acid, mannitol, and sucrose. The strongest biofilms by far, however, were formed with casamino acids as the source of carbon. This may be due to signaling considerations, as amino acids are present in plant exudates [45], or energetic considerations, because these cultures have a lower anabolic load. It should be noted here that some components of the casein hydrolysate might be used as a nitrogen source in this instance. Simultaneous growth experiments suggest that maleic acid, maltose, sucrose, and sodium benzoate are poor growth substrates in this particular format, although strong growth on these substrates was evident in well aerated culture tubes under identical nutrient conditions. This is the likely explanation for the low biofilm formation with these substrates (Fig 8B). In culture conditions under shear, filamentous forms were frequently observed, suggesting a developmental response to this physical stress. The larger scale structure of a biofilm under continuous nutrient flow developed similarly in our two sheared bioreactors, with an early phase of "pioneer" cells attaching to the surface, and microcolony formation (Fig 9B, Fig 10A, B). As the film developed further with input of nutrients, the honeycomb structure frequently observed in other biofilms [46] is apparent (Fig 10C, F). Our data support the notion of exopolysaccharide (eps) production as a primary consideration in biofilm productivity, with some potential staining of eps present in our static biofilm experiments (Fig 9A). This critical role of eps has been identified in numerous other systems (for review see [26]), and is reaffirmed in this work. This bacterium forms robust biofilms on abiotic surfaces under diverse culture conditions in the laboratory, consistent with the production of a profuse, sticky matrix. Further genetic work (Pehl et al, manuscript in preparation) has shown that putative LPS/eps synthesis genes are important in this phenotype.

## Conclusion

In this work we have established culture techniques for studying coordinated surface behaviors in the ubiquitous soil bacterium *Variovorax paradoxus*. We have shown that swarming motility in this organism is robust and varies sharply with a number of nutrient and media conditions. Similarly, we have shown that biofilms are formed by this organism in various culture media, and that these biofilms are likewise affected by nutrient conditions, but also by shear conditions. These studies will form the basis for future genetic studies of this strain of *V. paradoxus*, and will help us understand the role of this bacterium in the soil environment.

## Authors' contributions

WDJ performed many of the swarming assays and the biofilm nutrient dependence studies. MJP performed the swarming assays to examine carbon source dependence. GAG performed the assays to examine swarming on various nitrogen sources. PMO performed the static and continuous biofilm chamber experiments, as well as many swarming assays. PMO wrote the manuscript, with contributions from the three other authors. All authors have read and approved the final manuscript.

## Supplementary Material

Additional file 1 ***Variovorax paradoxus *EPS swarming time-lapse video**. This is a video of *V. paradoxus *EPS swarming on FW-succinate-NH_4_Cl medium take 18 h post inoculation. 2 h time lapse, 3 m between frames. Click here for file

## References

[B1] Willems A Ley JD Gillis M Kersters K Comamonadaceae, a new family encompassing the Acidovorans rRNA complex, including Variovorax paradoxus gen. nov., comb. nov., for Alcaligenes paradoxus (Davis 1969) Int J Syst Bacteriol 1991 41 445–450

[B2] Trusova MY Gladyshev MI Phylogenetic diversity of winter bacterioplankton of eutrophic siberian reservoirs as revealed by 16S rRNA gene sequence Microb Ecol 2002 44 3 252 259 12209253 10.1007/s00248-002-2020-1

[B3] Smith D Alvey S Crowley DE Cooperative catabolic pathways within an atrazine-degrading enrichment culture isolated from soil FEMS Microbiol Ecol 2005 53 2 265 273 16329946 10.1016/j.femsec.2004.12.011

[B4] Nishino SF Spain JC Biodegradation of 3-nitrotyrosine by Burkholderia sp. strain JS165 and Variovorax paradoxus JS171 Appl Environ Microbiol 2006 72 2 1040 1044 16461647 10.1128/AEM.72.2.1040-1044.2006 PMC1392975

[B5] Leadbetter JR Greenberg EP Metabolism of acyl-homoserine lactone quorum-sensing signals by Variovorax paradoxus J Bacteriol 2000 182 24 6921 6926 11092851 10.1128/jb.182.24.6921-6926.2000 PMC94816

[B6] Maimaiti J Zhang Y Yang J Cen YP Layzell DB Peoples M Dong Z Isolation and characterization of hydrogen-oxidizing bacteria induced following exposure of soil to hydrogen gas and their impact on plant growth Environ Microbiol 2007 9 2 435 444 17222141 10.1111/j.1462-2920.2006.01155.x

[B7] Anesti V McDonald IR Ramaswamy M Wade WG Kelly DP Wood AP Isolation and molecular detection of methylotrophic bacteria occurring in the human mouth Environ Microbiol 2005 7 8 1227 1238 16011760 10.1111/j.1462-2920.2005.00805.x

[B8] Shapiro JA The significances of bacterial colony patterns Bioessays 1995 17 7 597 607 7646482 10.1002/bies.950170706

[B9] Spormann AM Gliding motility in bacteria: insights from studies of Myxococcus xanthus Microbiol Mol Biol Rev 1999 63 3 621 641 10477310 10.1128/mmbr.63.3.621-641.1999 PMC103748

[B10] Henrichsen J Bacterial surface translocation: a survey and a classification Bacteriol Rev 1972 36 4 478 503 4631369 10.1128/br.36.4.478-503.1972 PMC408329

[B11] Matz C McDougald D Moreno AM Yung PY Yildiz FH Kjelleberg S Biofilm formation and phenotypic variation enhance predation-driven persistence of Vibrio cholerae Proc Natl Acad Sci USA 2005 102 46 16819 16824 16267135 10.1073/pnas.0505350102 PMC1283802

[B12] Boles BR Thoendel M Singh PK Self-generated diversity produces "insurance effects" in biofilm communities Proc Natl Acad Sci USA 2004 101 47 16630 16635 15546998 10.1073/pnas.0407460101 PMC528905

[B13] Rice SA Koh KS Queck SY Labbate M Lam KW Kjelleberg S Biofilm formation and sloughing in Serratia marcescens are controlled by quorum sensing and nutrient cues J Bacteriol 2005 187 10 3477 3485 15866935 10.1128/JB.187.10.3477-3485.2005 PMC1111991

[B14] Coetzee JN Deklerk HC Effect Of Temperature On Flagellation, Motility And Swarming Of Proteus Nature 1964 202 211 212 14156320 10.1038/202211b0

[B15] Kearns DB Losick R Swarming motility in undomesticated Bacillus subtilis Mol Microbiol 2003 49 3 581 590 12864845 10.1046/j.1365-2958.2003.03584.x

[B16] Givskov M Ostling J Eberl L Lindum PW Christensen AB Christiansen G Molin S Kjelleberg S Two separate regulatory systems participate in control of swarming motility of Serratia liquefaciens MG1 J Bacteriol 1998 180 3 742 745 9457883 10.1128/jb.180.3.742-745.1998 PMC106947

[B17] Overhage J Lewenza S Marr AK Hancock RE Identification of genes involved in swarming motility using a Pseudomonas aeruginosa PAO1 mini-Tn5-lux mutant library J Bacteriol 2007 189 5 2164 2169 17158671 10.1128/JB.01623-06 PMC1855721

[B18] Kaiser D Bacterial swarming: a re-examination of cell-movement patterns Curr Biol 2007 17 14 561 570 10.1016/j.cub.2007.04.050 17637359

[B19] Wang Q Frye JG McClelland M Harshey RM Gene expression patterns during swarming in Salmonella typhimurium: genes specific to surface growth and putative new motility and pathogenicity genes Mol Microbiol 2004 52 1 169 187 15049819 10.1111/j.1365-2958.2003.03977.x

[B20] Connelly MB Young GM Sloma A Extracellular proteolytic activity plays a central role in swarming motility in Bacillus subtilis J Bacteriol 2004 186 13 4159 4167 15205417 10.1128/JB.186.13.4159-4167.2004 PMC421602

[B21] Kim W Surette MG Prevalence of surface swarming behavior in Salmonella J Bacteriol 2005 187 18 6580 6583 16159794 10.1128/JB.187.18.6580-6583.2005 PMC1236657

[B22] Kohler T Curty LK Barja F van Delden C Pechere JC Swarming of Pseudomonas aeruginosa is dependent on cell-to-cell signaling and requires flagella and pili J Bacteriol 2000 182 21 5990 5996 11029417 10.1128/jb.182.21.5990-5996.2000 PMC94731

[B23] Shrout JD Chopp DL Just CL Hentzer M Givskov M Parsek MR The impact of quorum sensing and swarming motility on Pseudomonas aeruginosa biofilm formation is nutritionally conditional Mol Microbiol 2006 62 5 1264 1277 17059568 10.1111/j.1365-2958.2006.05421.x

[B24] Steil L Hoffmann T Budde I Volker U Bremer E Genome-wide transcriptional profiling analysis of adaptation of Bacillus subtilis to high salinity J Bacteriol 2003 185 21 6358 6370 14563871 10.1128/JB.185.21.6358-6370.2003 PMC219388

[B25] Wang Q Suzuki A Mariconda S Porwollik S Harshey RM Sensing wetness: a new role for the bacterial flagellum Embo J 2005 24 11 2034 2042 15889148 10.1038/sj.emboj.7600668 PMC1142604

[B26] Hall-Stoodley L Costerton JW Stoodley P Bacterial biofilms: from the natural environment to infectious diseases Nat Rev Microbiol 2004 2 2 95 108 15040259 10.1038/nrmicro821

[B27] Davey ME O'Toole GA Microbial biofilms: from ecology to molecular genetics Microbiol Mol Biol Rev 2000 64 4 847 867 11104821 10.1128/mmbr.64.4.847-867.2000 PMC99016

[B28] Tart AH Wozniak DJ Shifting paradigms in Pseudomonas aeruginosa biofilm research Curr Top Microbiol Immunol 2008 322 193 206 18453277 10.1007/978-3-540-75418-3_9

[B29] Spormann AM Physiology of microbes in biofilms Curr Top Microbiol Immunol 2008 322 17 36 18453270 10.1007/978-3-540-75418-3_2

[B30] Dunny GM Brickman TJ Dworkin M Multicellular behavior in bacteria: communication, cooperation, competition and cheating Bioessays 2008 30 4 296 298 18348154 10.1002/bies.20740

[B31] Jones BV Mahenthiralingam E Sabbuba NA Stickler DJ Role of swarming in the formation of crystalline Proteus mirabilis biofilms on urinary catheters J Med Microbiol 2005 54 Pt 9 807 813 16091430 10.1099/jmm.0.46123-0

[B32] Davey ME Costerton JW Molecular genetics analyses of biofilm formation in oral isolates Periodontol 2000 2006 42 13 26 16930303 10.1111/j.1600-0757.2006.00052.x

[B33] Ryu JH Kim H Beuchat LR Attachment and biofilm formation by Escherichia coli O157:H7 on stainless steel as influenced by exopolysaccharide production, nutrient availability, and temperature J Food Prot 2004 67 10 2123 2131 15508620 10.4315/0362-028x-67.10.2123

[B34] Mohamed JA Huang DB Biofilm formation by enterococci J Med Microbiol 2007 56 Pt 12 1581 1588 18033823 10.1099/jmm.0.47331-0

[B35] Yarwood JM Bartels DJ Volper EM Greenberg EP Quorum sensing in Staphylococcus aureus biofilms J Bacteriol 2004 186 6 1838 1850 14996815 10.1128/JB.186.6.1838-1850.2004 PMC355980

[B36] Aballay A Drenkard E Hilbun LR Ausubel FM Caenorhabditis elegans innate immune response triggered by Salmonella enterica requires intact LPS and is mediated by a MAPK signaling pathway Curr Biol 2003 13 1 47 52 12526744 10.1016/s0960-9822(02)01396-9

[B37] Danhorn T Fuqua C Biofilm formation by plant-associated bacteria Annu Rev Microbiol 2007 61 401 422 17506679 10.1146/annurev.micro.61.080706.093316

[B38] Brewster JD A simple micro-growth assay for enumerating bacteria J Microbiol Methods 2003 53 1 77 86 12609726 10.1016/s0167-7012(02)00226-9

[B39] Caiazza NC Shanks RM O'Toole GA Rhamnolipids modulate swarming motility patterns of Pseudomonas aeruginosa J Bacteriol 2005 187 21 7351 7361 16237018 10.1128/JB.187.21.7351-7361.2005 PMC1273001

[B40] Ingham CJ Ben Jacob E Swarming and complex pattern formation in Paenibacillus vortex studied by imaging and tracking cells BMC Microbiol 2008 8 36 18298829 10.1186/1471-2180-8-36 PMC2268691

[B41] Aragno M Walther-Mauruschat A Mayer F Schlegel HG Micromorphology of Gram-negative hydrogen bacteria. I. Cell morphology and flagellation Arch Microbiol 1977 114 2 93 100 410385 10.1007/BF00410769

[B42] Matsuyama T Bhasin A Harshey RM Mutational analysis of flagellum-independent surface spreading of Serratia marcescens 274 on a low-agar medium J Bacteriol 1995 177 4 987 991 7860610 10.1128/jb.177.4.987-991.1995 PMC176693

[B43] Chen BG Turner L Berg HC The wetting agent required for swarming in Salmonella enterica serovar typhimurium is not a surfactant J Bacteriol 2007 189 23 8750 8753 17905988 10.1128/JB.01109-07 PMC2168935

[B44] Gibbs KA Urbanowski ML Greenberg EP Genetic determinants of self identity and social recognition in bacteria Science 2008 321 5886 256 259 18621670 10.1126/science.1160033 PMC2567286

[B45] Bais HP Weir TL Perry LG Gilroy S Vivanco JM The role of root exudates in rhizosphere interactions with plants and other organisms Annual Review of Plant Biology 2006 57 233 266 16669762 10.1146/annurev.arplant.57.032905.105159

[B46] Fux CA Costerton JW Stewart PS Stoodley P Survival strategies of infectious biofilms Trends Microbiol 2005 13 1 34 40 15639630 10.1016/j.tim.2004.11.010

